# Association of Use of Online Symptom Checkers With Patients’ Plans for Seeking Care

**DOI:** 10.1001/jamanetworkopen.2019.18561

**Published:** 2019-12-27

**Authors:** Aaron N. Winn, Melek Somai, Nicole Fergestrom, Bradley H. Crotty

**Affiliations:** 1 Medical College of Wisconsin, Milwaukee

## Abstract

This cross-sectional study examines the association of patient use of a free online symptom checker tool with patient plans for seeking medical care.

## Introduction

Online triage tools are increasingly being adopted in health care to aid patients in identifying the appropriate care level.^1^ However, there is a lack of empirical evidence on how patients use virtual triage and whether these tools influence care-seeking behavior.^1,2,3^ Using data from a free online triage tool, we describe the common symptoms queried by users and analyze whether the tool was associated with the level of care that patients intended to seek.

## Methods

This cross-sectional study used data from patient encounters using Buoy Health, a freely available virtual triage chatbot. The tool is based on conversational medical interviewing.^4^ A user is prompted to type 1 or more concerns or symptoms and minimal demographic information (age and sex) and then respond to questions. At the end of the process, the chatbot provides up to 3 possible explanations with recommended levels of care. We classified patients’ chief concerns into categories based on organ system, concern, and location. The data include completed encounters in which preencounter and postencounter intention data were provided for 158 083 encounters, representing 12% of encounters from March 27, 2018, through December 18, 2018. For each encounter, users were prompted for their intended level of care at the beginning (preencounter intent) and at the end of the encounter (postencounter intent). The preencounter intent and postencounter intent levels of care were ranked by relative urgency, from watch and wait (level 0) to emergency department (level 7). The Medical College of Wisconsin institutional review board considered this study using a deidentified data set non–human participants research. We used the Strengthening the Reporting of Observational Studies in Epidemiology (STROBE) reporting guideline to guide our reporting.

We used descriptive statistics to examine intended behavior by comparing patients’ preencounter and postencounter intent level of care. To assess the tool’s association with care seeking, we removed uncertain responses and examined whether their intended level of care increased or decreased. We used 2-sided χ^2^ tests to examine whether associations were statistically significant. A *P* value of .05 was considered statistically significant.

## Results

The mean (SD) participant age was 40 (16.5) years and 122 929 of 158 083 encounters (78%) involved female patients. The 158 083 encounters resulted in 281 165 reported symptoms, with 204 958 symptoms having identifiable organ systems and 279 948 having identifiable concern types. The most common organ system concerns were related to reproductive (37 669 of 204 958 reported symptoms [18%]), general (34 120 of 204 958 reported symptoms [17%]), and gastrointestinal (30 994 of 204 958 reported symptoms [15%]) systems. The most common symptom types were pain (122 363 of 279 948 reported symptoms [44%]), abnormal functioning (62 949 of 279 948 reported symptoms [22%]), and discharge (21 622 of 279 948 reported symptoms [8%]). The most common preencounter intended level of care was primary care (73 546 encounters [47%]), followed by uncertain (53 649 encounters [34%]), urgent (14 928 encounters [9%]), and emergency (14 813 encounters [9%]). We found that the proportion of patients who were uncertain about the level of care decreased from 34% to 21% (difference, 13.4%; 95% CI, 13.1%-13.7%; *P* < .001). Among patients who chose preencounter and postencounter intended level of care (ie, did not select “uncertain”), we found that in 28 961 interactions (32%), patients reduced the urgency of their intended level of care; in 58 844 (65%), urgency remained the same; and in 3243 (4%), urgency increased (χ^2^_15_ = 77936.6960; *P* < .001) (Figure).

**Figure.**
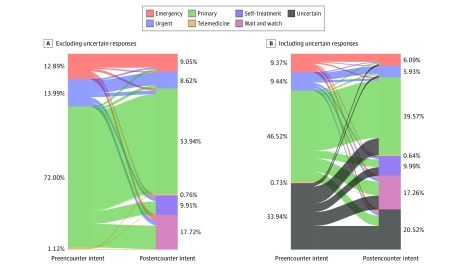
Planned Level of Care Before and After Use of an Online Symptom Checker The 158 083 patient interactions with the online triage tool do not represent a unique patient count; rather, if patients used the tool twice, they would be included twice in the data. When declaring the preencounter intent and postencounter intent planned level of care, there were 2 additional choices (self-treatment and watch and wait) for the postencounter intent planned level of care.

## Discussion

In this data set of more than 150 000 patient interactions with an online triage tool, the urgency of patients’ intended level of care decreased in more than one-quarter of the cases and increased in approximately 1 in 20 cases, with the remaining patients remaining at the same level. The study suggests that virtual triage tools are associated with patients’ intended behavior when seeking care based on triage questions. Reduced urgency of intended level of care for many interactions is different from many other direct-to-consumer telehealth solutions, which have reported the same or an increased demand for care services.^5^

This preliminary research has several limitations. We only included patient interactions that provided postencounter intention data. We only have information within the context of patient-tool interactions and were not able to observe whether users had multiple interactions, whether behavior was affected, or the appropriateness of the triage results.

Future research should link patients’ use of such online tools with health care records to understand how patients use online tools in tandem with clinicians to manage their health.
